# The Influence of Burnout on Patient Safety Management Activities of Shift Nurses: The Mediating Effect of Compassion Satisfaction

**DOI:** 10.3390/ijerph182212210

**Published:** 2021-11-20

**Authors:** I Seul Ryu, JaeLan Shim

**Affiliations:** 1 Department of Nursing, Dongguk University Hospital, Gyeongju 38067, Korea; carpediemris@naver.com; 2 College of Nursing, Dongguk University, Gyeongju 38066, Korea

**Keywords:** patient safety, burnout, compassion satisfaction, shift nurse

## Abstract

This study aims to investigate the levels of burnout, compassion satisfaction (CS), and patient safety management activities (PSMA) among nurses on shift work in general hospitals and to examine the mediating effect of CS on the relationship between burnout and PSMA. This was a descriptive-correlational study, conducted with a convenience sample of 301 nurses from four general hospitals. Data were collected from 1 August to 4 September 2021, using self-report questionnaires. Data were analyzed using the *t*-test, analysis of variance, Tukey test, Pearson’s correlation coefficient, and multiple regression analyses. Mediation analysis was performed according to the Baron and Kenny, and Sobel Tests. Significant relationships were found between shift nurses’ burnout and CS (r = −0.66, *p* < *0*.001), burnout and PSMA (r = −0.32, *p* < 0.001), and PSMA and CS (r = 0.32, *p* < 0.001). Compassion satisfaction showed partial mediating effects on the relationship between burnout and PSMA (Z = −3.21, *p* = 0.001). The higher the burnout of shift-working nurses, the lower the patient safety management activity. Therefore, an effective support system at the organizational level as well as individual efforts are necessary to enhance CS and reduce burnout of shift-working nurses.

## 1. Introduction

Patient safety is the first underlying principle in the provision of medical services that all members of a hospital should have in common [1]. In a recent trend of patient safety incidents in Korea, 9250 cases were reported in 2018, 11,953 cases in 2019, and in 2020, the monthly average was more than 100 cases, amounting to 13,919 cases in the year. This trend shows a gradual increase of approximately 1.5 times in the last three years, and approximately 50% of these incidents have reportedly been caused by inadequate nursing care [2].

Nurses who are responsible for quick and sensitive detection and responses regarding patient safety care during their close interactions with patients 24 h a day are mostly subject to shift work. This often leads to fatigue and disturbances of their sleep, thereby decreasing the accuracy and efficiency of their nursing services. This may increase the risk of in-hospital safety incidents, such as medication errors, patient identification errors, inappropriate operation of medical devices, and injury from injection syringes. Such incidents may have adverse effects on the health and lives of patients [3,4,5]. Therefore, there has been an increasing emphasis on the importance of patient safety management activities (PSMA) of shift nurses in the interest of patient safety.

According to a recent study, a higher proportion of nurses experienced burnout than other healthcare workers [6,7]. Moreover, higher levels of burnout were reported among shift-working nurses [8]. The burnout experience of shift-working nurses not only has a damaging impact on their physical and mental health but also leads to loss of motivation for work and indifference toward patients [9], causing more patient safety incidents, such as falls and medication errors, which adversely affect PSMA [10]. Additionally, in a prior study analyzing the findings of 21 systematic literature reviews and meta-analyses, it was reported that a high level of burnout among health professionals worsens patient safety [11], confirming the importance of managing burnout in shift-working nurses.

While providing nursing care for their patients, nurses experience compassion satisfaction (CS), a pleasant emotion that comes from having the ability to help others [12]. CS is the variable that has the greatest impact on burnout, which is a negative effect and has been reported to serve as a buffer or moderator of burnout [13,14]. A close relationship is formed with patients through compassion, which leads to the feeling of positive emotions when providing nursing care by listening to the needs of the patients and eliciting interest from patients; this combines with nurses’ self-esteem and professionalism and enhances their job satisfaction and performance [15,16,17]. Furthermore, CS induces active coping among nurses regarding promoting patient safety through sensitive responses to the condition of the patients [18], which may have a direct impact on patient nursing outcomes.

Research has been conducted on burnout and patient safety culture as predictors of PSMA among shift nurses. Regarding the predictors for PSMA confirmed in existing research, it has been reported that PSMA increases with lower levels of burnout and with increasing job satisfaction of patient safety culture as well as a high level of patient safety management activities [19]. However, there have been limited studies on the association between CS and burnout as a predictor of PSMA. Therefore, this study aims to investigate the levels of burnout, CS, and PSMA among nurses on shift work in general hospitals and to examine the mediating effect of CS on the relationship between burnout and PSMA.

The detailed objectives are outlined as follows:

- Investigating the differences in burnout, CS, and PSMA according to the general and job-related characteristics of nurses;
- Investigating the correlation between burnout, CS, and PSMA of the nurses; and
- Examining the effect of nurses’ burnout on PSMA and the mediating effect of CS between these two variables.

## 2. Materials and Methods

### 2.1. Study Design

This study adopted a descriptive cross-sectional survey.

### 2.2. Study Participants

The participants in this study were 301 shift-working nurses in general hospitals comprising more than 300 beds, located in a city in Korea. The inclusion criteria for participants are those who gave voluntary consent to participate and shift-working nurses who have practiced direct nursing with a work experience of more than six months. These were based on the results of a previous study [20], which reported that it requires six months on average for a new nurse to be able to independently perform a task. Nurses who were head nurses or higher and did not practice direct nursing were excluded from the study. The sample size was obtained using G*Power 3.1.9.2 (University of Dusseldorf, Germany). For the variables to be analyzed in this study, the sample size required for 14 predictive variables, with a significance level of 0.05, median effect size of 0.15, and power of 0.95, was calculated as 194. Therefore, the number of participants in this study satisfied the sample size required for regression analysis. Of the 330 copies of the distributed questionnaire, 301 copies (91%) were completed and returned for data analysis; 29 copies were excluded due to incomplete data. Informed consent was obtained from all participants involved in the study.

### 2.3. Instruments

#### 2.3.1. Patient Safety Management Activities

To assess PSMA, the patient safety management activities measure developed by Lee [21] through a literature review on standards related to patient safety management, including the standard specified by the Joint Commission on Accreditation of Healthcare Organization (JCAHO), was used. This scale consists of 46 items and 10 sub-items. The tools comprise 7 items for patient identification, 6 items for communication, 3 items for oral prescription management, 7 items for medication management, 4 items for surgery and procedure management, 3 items for environmental management, 3 items for infection prevention, 3 items for fall prevention, 3 items for bedsore prevention, and 7 items for crisis management.

This scale consists of a 5-point Likert scale, with 1 point indicating “Not at all” and 5 points indicating “Very true.” The higher the score, the better the patient safety management activities performed. Regarding the reliability of the scale during its development, Cronbach’s α was 0.92 in the original research by Lee [21], and in this study, Cronbach’s α was 0.96.

#### 2.3.2. Burnout

To assess the burnout of shift nurses, the Korean version of the PROQOLS Version 5 (Professional Quality of Life Scale; compassion satisfaction/fatigue subscale—version 5), which is a modified and improved version by Stamm [12] of the original Compassion Satisfaction/Fatigue Self-Test for Helpers, developed by Figley [22], was used. This scale is categorized into compassion satisfaction, burnout, and secondary traumatic stress, and consists of 30 items with 10 items for each category. From the 30 items, the 10 items on burnout were used for measurement. Each item is assessed on a 5-point Likert scale, with 1 point indicating “Not at all” and 5 points indicating “Very true.” Regarding the reliability of burnout during the development of this scale, Cronbach’s α was 0.75 in the original research by Stamm, and in this study, Cronbach’s α was 0.76.

#### 2.3.3. Compassion Satisfaction

To assess CS, the Korean version of the PROQOLS Version 5 was used. From the 30 items, the 10 items on CS were used for assessment. Each item is assessed on a 5-point Likert scale, with 1 point indicating “Not at all” and 5 points indicating “Very true.” Regarding the reliability of burnout during the development of this scale, Cronbach’s α was 0.88 in the original research by Stamm, and in this study, Cronbach’s α was 0.93.

#### 2.3.4. General and Job-Related Characteristics of Participants

The general characteristics include age, gender, marital status, religious status, education level, regular exercise status, subjective health status, working period, position, work department, number of night shifts per month, and subjective job stress level.

### 2.4. Ethical Consideration

This study was approved by the Institutional Review Board of D University (IRB No. DGU IRB 20190018-02). The purpose of the study and the contents of the questionnaire were explained, and the participants were informed that there would be no disadvantages if they refuse to respond or withdraw from the study. Finally, signed consent forms were collected from those who voluntarily expressed their wish to participate in the study.

### 2.5. Data Collection Method

The data were collected for the period from 1 August 2021 to 4 September 2021. The study targeted shift nurses who had been working at general hospitals in Korea for more than six months, and according to the prescribed procedure of the hospitals, the researcher directly visited the head of the nursing division, explained the purpose of the study and the contents of the questionnaire, and obtained permission to conduct the study. The questionnaire was then distributed by the researchers. Before data collection, the researchers reviewed and adjusted any item in the questionnaire that might elicit a favorable answer, including binary response formats such as “Yes/No,” “True/False,” and “Agree/Disagree.” In addition, we introduced measurements to help the participants focus. This method raises understanding and removes the likelihood that they will skim over the content of the question or fail to understand what is being asked.

### 2.6. Data Analysis

The collected data were analyzed using IBM SPSS/WIN 25.0 (IBM Corp. Armonk, NY, USA). The general characteristics and job-related characteristics of the participants were represented as frequencies, percentages, means, and standard deviations; the levels of burnout, PSMA, and CS were represented as means and standard deviations. Differences in PSMA according to the participants’ general and job-related characteristics were analyzed using the *t*-test and ANOVA, with, as a post-hoc test, Tukey HSD (Honest Significant Difference), which is the best for all-possible pairwise comparisons when sample sizes are unequal, or confidence intervals are needed [23].

The correlations between burnout, PSMA, and CS were analyzed using Pearson’s correlation coefficients. Multiple regression analysis was conducted to examine the mediating effect of CS on the relationship between burnout and PSMA. To validate the mediator, a stepwise method proposed by Baron and Kenny [24] was used, and simple regression analysis was performed to determine the effect of the independent variable on the mediator in Step 1 and to determine the effect of the independent variable on the dependent variables in Step 2. To establish the mediating effect in Step 3, the effects of the independent variable and mediator on the dependent variables were confirmed through a hierarchical multiple regression analysis. The significance of the mediating effect was verified by the Sobel test of mediation effect (Z) [25].

## 3. Results

### 3.1. General Characteristics of Participants

Table 1 shows the general characteristics of the subjects. The average age of the participants was 28.53 (±5.94) years. The age group was 25–29 years old with 39.9%, and most of the participants were female (94.0%). A total of 79.1% of the subjects were single people and participants without religious affiliation (60.5%) and had a bachelor’s degree or higher (59.1%). More than half of the subjects exercised regularly (57.1%), and their subjective health status was moderate or poor (73.4%).

In terms of job-related characteristics, those with less than two years of clinical experience accounted for 38.0%, and most of the participants (95.7%) were staff nurses. A total of 30.2% were internal medicine ward workers, 49.8% were participants who work 7–8 night duties per month, and 96.0% were participants who felt subjective job stress was moderate to heavy (Table 1).

### 3.2. Differences in Burnout, CS, and PSMA According to General and Job-Related Characteristics of Shift-Working Nurses

As a result of Levene’ s test of variables of burnout, PSMA, and CS, the test statistic (F = 0.09, *p* = 0.914) showed that the *p*-value was greater than the significance level (0.05), which satisfies the assumption of equal variance. The level of variance was constant across the sample; hence, an ANOVA test was performed.

Table 1 shows the degree of burnout, CS, and PSMA according to the general and job-related characteristics of the participants. The degree of burnout of the participants was higher in those without religion (t = 2.00, *p* = 0.047), those with a college or bachelor’s degree (F = 3.14, *p* = 0.045), and those who did not exercise. It was higher in the case of not (t = 3.41, *p* = *0*.001). Subjective health status was poor (F = 10.96, *p* < 0.001). Internal medicine ward workers (F = 3.13, *p* = 0.009) and participants with high subjective job stress (F = 28.67, *p* < 0.001) showed a high burnout score. The degree of CS was higher in married people (t = −2.38, *p* = 0.018) than in single people, participants with religious affiliation (t = −3.08, *p* = *0*.002), and in those with a master’s degree or higher (t = 3.39, *p* = 0.035). Participants with good subjective health (F = 3.61, *p* = 0.028), charge nurses rather than staff nurses (t = −2.05, *p* = 0.042), and participants with less subjective job stress (F = 5.13, *p* = 0.006) showed high CS. The PSMA level was found to be higher in participants who exercised regularly (t = −2.36, *p* = 0.019) and those who had less subjective job stress (F = 6.01, *p* = 0.003) (Table 1).

### 3.3. Participant’s Burnout, CS, and PSMA Level

Table 2 shows the burnout, CS, and PSMA levels of the participants.

**Figure 1:**
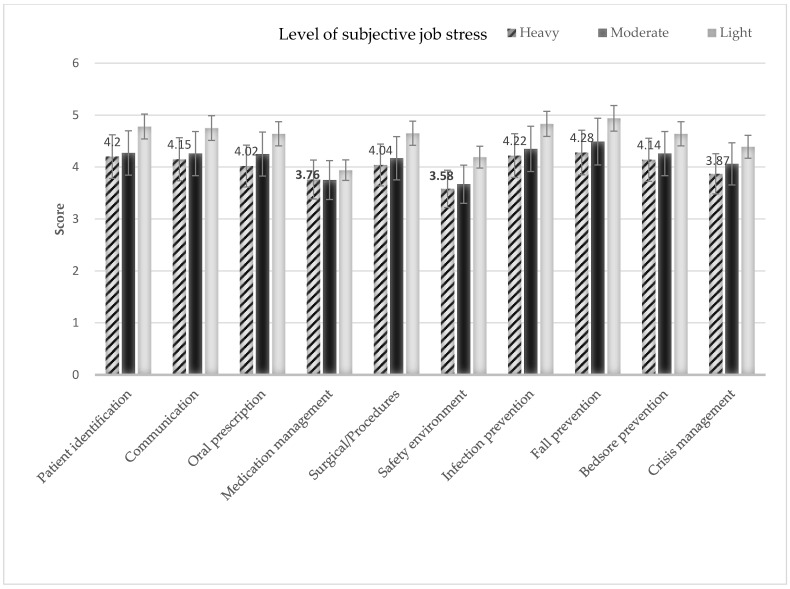
The scores for the sub-areas of PSMA according to the subjective job stress level.

The scores for the sub-areas of PSMA according to subjective job stress are shown in Figure 1 (converted to a 5-point scale), and the domains with heavy job stress and low PSMA scores were medication management and safety environment management (Figure 1).

### 3.4. CorrelationsamongBurnout, CS, and PSMA

The correlations among burnout, CS, and PSMA of the participants are presented in Table 3. Burnout and CS showed a significant negative correlation (r = −0.66, *p* < 0.001). PSMA was negatively correlated with burnout (r = −0.32, *p* < 0.001) and positively correlated with CS (r = 0.32, *p* < 0.001).

### 3.5. Mediating Effect of CS on the Relationship between Burnout and PSMA

Multiple regression analysis was performed to examine the mediating effect of CS on the relationship between burnout and PSMA. Before testing the mediating effect, we first examined whether the assumption of regression analysis was satisfied. The Durbin Watson index for autocorrelation between the variables was from 1.67 to 1.97, which is close to 2, indicating independence between the variables. The variance inflation factor was in the range of 1.00–1.77, which is less than 10, indicating that there is no multicollinearity between independent variables.

In this study, the three-step analysis proposed by Baron and Kenny [24] was performed to verify the mediating effect of burnout on PSMA through CS.

**Figure 2:**
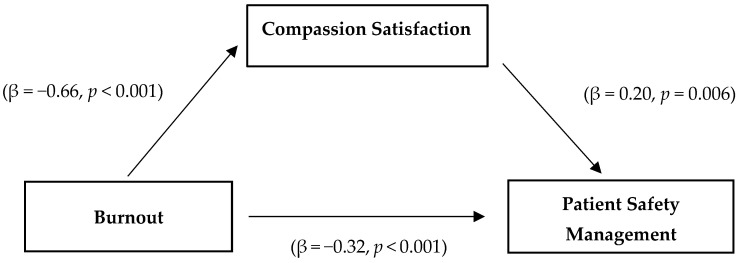
Mediating effect of compassion satisfaction on the relationship between burnout and patient safety management activities.

As a result of Step 1 of the analysis, burnout, an independent variable, had a significant effect on the mediator CS (β = −0.66, *p* < *0*.001), with an explanatory power of 43%. As a result of Step 2 of the analysis, burnout, an independent variable, had a significant effect on PSMA, a dependent variable (β = −0.32, *p* < *0*.001), with an explanatory power of 10%. As a result of Step 3, by setting burnout and CS as independent variables and PSMA as a dependent variable for analysis, burnout (β = −0.18, *p* = 0.011) and CS (β = 0.20, *p* = 0.006) were shown to have a significant effect on PSMA. That is, when CS was used as a mediator in Step 3, burnout had a significant impact on PSMA, but the regression coefficient (β) decreased from −0.32 in Step 2 to −0.18, indicating that CS served as a partial mediator. The explanatory power of these variables for PSMA was 12%. In the relationship between burnout and PSMA, the results of the Sobel test performed for the significance testing of the mediating effect of CS were significant (Z = −3.21, *p* = 0.001) (Figure 2). Therefore, the results of this study verify that there is a mediating effect of CS in the process of burnout affecting PSMA (Table 4).

## 4. Discussion

This study was performed to determine the mediating effect of CS on the effect of burnout of shift-working nurses in general hospitals on PSMA and to utilize the results as basic reference data to enhance the CS and PSMA of shift nurses.

In this study, the level of PSMA of shift nurses was 4 out of 5 on average (converted to a 5-point scale), which is a lower score in comparison to 4.38 points in a prior study of university hospital nurses [26] and 4.08 points for nurses in small- and medium-sized hospitals [27]; however, it is a higher score when compared to 3.32 points [28], the PSMA score of a previous study conducted with nurses in a general hospital of a similar size to the participating hospitals in this study. These differences in the PSMA score are thought to be due to the differences in the sizes, characteristics, and systems of hospitals, indicating the need for a large-scale multicenter study in the future. In addition, the studies that have been compared with this study have all used different instruments to assess PSMA, and it is thought that the development of a more standardized instrument for the assessment of PSMA is necessary. Examining the scores by the sub-areas of PSMA, fall-prevention activity scored the highest at 4.37 points, followed by infection-prevention activity at 4.29 points and patient identification at 4.24 points, and medication nursing and safety environment scored relatively low at 3.76 and 3.63 points, respectively. From the results, although fall-prevention activity showed high scores among the sub-areas of PSMA, more than 50% of patient safety incidents were related to falls [29], which indicates that the active training intervention of nurses for fall prevention is required. The sub-area with the lowest score was safety environment, and this is thought to be because safety environment is activity related to the provision of safe systems in hospitals; thus, the shift nurses showed a lower level of interest compared to other activities related to the provision of direct nursing. In addition, although medication nursing accounts for a significant part of nursing work, the results show that there is a lower than average level of medication-related PSMA, indicating that the probability of medication errors is still high. The results reveal that there is a pressing need for further efforts to ensure safe medication nursing. The accurate administration of medication is an important responsibility of nurses and accounts for a significant part of nursing work. Despite the high probability of medication errors [30,31], the responsibility for the medication error usually lies on the individual nurse, and there is an organizational atmosphere regarding the reporting of the medication error to the nurse manager or exposing the information outside [32]. Therefore, rather than pointing out medication errors of individual nurses that have already occurred, concerted efforts should be made to establish a safe organizational system of medication or to create an organizational atmosphere that does not hinder the reporting of errors. However, since medication is a nursing intervention directly related to the life of patients and an essential responsibility of nurses, there should be continuous individual efforts made to prevent medication errors.

The total score representing the level of burnout of shift nurses in this study is 29.89 points. This score is similar to 29.3 points [33] of clinical nurses measured using the same instrument in a previous study. Compared to the average burnout scores of 2.37–2.5 points [34,35,36] reported in studies on clinical nurses overseas, the average score of shift nurses’ burnout in this study is 2.96 points, indicating that the nurses were experiencing high levels of burnout. Lack of time and resources are the main causes of nurses’ burnout. Time pressure regards the state in which the nurses feel pressure to resolve problems in insufficient time, and it has been reported that nurses with perceived time pressure have little job satisfaction, which can lead to the worsening of burnout [37]. The ratio of nursing workforce supply in service in Korea is 5.9 per 1000 people, and compared to the average ratios of 9.1 in Organization for Economic Co-operation and Development (OECD) and 18 for Switzerland, the supply of the clinical nursing workforce is in a considerable shortage [38].

In particular, it is thought that Korean nurses have little job satisfaction due to the heavy workload of nursing activities that must be performed within a given time, aggravating burnout. Therefore, to mitigate the burnout of nurses, non-nursing-related work performed by nurses should be identified, and improvement measures to reduce the workload of nurses and plans to reduce the burden of nurses should be established.

In this study, the total score of CS is 29.61 points. This is similar to the results of a previous study [39] that assessed the CS of clinical nurses using the same instrument. In the results of studies on overseas clinical nurses, the CS scores were in the range of 3.55–4.12 points, whereas the score of the shift nurses in this study is 2.96 points, showing a considerably lower level of CS. Compassion is an act of listening to the difficulties of patients, empathizing with, and making efforts to help patients [40]. Compared to the case of South Korea, overseas nurses have fewer patients in charge per nurse, which increases opportunities for interaction with patients and also increases direct nursing time, leading to higher CS scores. Therefore, active research to estimate the appropriate nursing manpower to improve the CS of shift nurses is necessary. In addition, the more positive the working environments where nurses perform their tasks are perceived, the higher the CS scores [41]; therefore, it is considered that appropriate manpower allocation and material support as well as the creation of a positive nursing work environments are necessary

As a result of examining the correlation between the major variables in this study, there was a significant negative correlation between burnout and PSMA among shift nurses. This was consistent with the results of previous studies [19,37] that reported a negative effect of higher levels of burnout on PSMA. In the case of burnout among shift nurses, those who spend the longest hours interacting with patients are found to be more tired due to an excessive workload [42]. Moreover, the experience of burnout leads to indifference to the patients and the psychological detachment of nurses from their work due to cynicism [43]. This shows that burnout has an adverse effect on the performance of effective nursing work, with a significant impact on patient safety. The excessive workload of shift nurses not only increases their job stress and fatigue but also leads to burnout, and therefore, their level of concentration in their work is reduced, which may increase the number of patient safety incidents [44]. Therefore, it is necessary to provide specific and detailed methods to actively cope with burnout at the organizational level, such as providing break times to prevent the accumulation and provide relieve of fatigue and, in particular, establishing an intervention program for the moderation and control of burnout among shift nurses.

In addition, there was a significant negative correlation between burnout and CS. This is consistent with the results of a previous study [45] conducted on clinical nurses, which confirmed that the higher the level of burnout, the lower the level of CS. CS is a psychological reward and emotional satisfaction obtained in the process of helping patients [12], and it can reduce the negative effects that may arise from providing nursing care for patients [46]. Therefore, since it can be seen that CS is a positive factor in maintaining emotional balance in relation to work, support at the level of total environment and organization as well as the efforts of nurses on a personal level are instrumental.

Moreover, the CS and PSMA of shift nurses showed a significant positive correlation, indicating that the higher the CS, the higher the PSMA. This indicates that nurses’ CS improves job satisfaction [16] and organizational commitment, and nurses with higher levels of organizational commitment have higher emotional stability, better communication with organizational members, and lower levels of physical and emotional burnout, thus showing a high level of PSMA performance [47]. Therefore, there is a pressing need for the establishment of strategies and methods to enhance CS and reduce burnout among nurses.

In this study, a partial mediating effect of CS was confirmed in the relationship between burnout and PSMA among shift nurses. That is, the higher the level of burnout of nurses, the lower the PSMA, indicating that the experience of high levels of burnout decreases PSMA through CS. This indicates that to achieve an improved level of PSMA of shift nurses, efficient improvement of jobs should be made to reduce work overload and mitigate burnout among nurses.

The burnout experienced by shift nurses, who interact closest to the patients, has a significant impact on patient safety, and in the process of the interactions, the positive CS experienced by nurses while providing nursing care for the patients is expected to reduce burnout, inducing active responses to and coping with PSMA. Therefore, further investigation is required for effective methods that can increase CS while reducing burnout.

This study had several limitations. As this research was conducted based on the convenience sampling of shift nurses from four general hospitals, the extracted sample consists of a questionnaire survey and includes the subjective opinions of the research participants. Therefore, it may not be considered an accurate representation of the population. Therefore, a large-scale randomized study should be conducted in the future. In addition, due to the current COVID-19 pandemic, nurses’ burnout may increase turnover intention [48], but nurses’ greater visibility, motivation to alleviate suffering, and social recognition may increase nurses’ compassion satisfaction level. Based on findings of a previous study [49], it is necessary to closely study the level of burnout, CS, and PSMA of nurses in line with the pandemic situation in the future.

Additionally, this study is a cross-sectional and correlational study that examined the mediating effect of CS on the relationship between burnout and PSMA among nurses; therefore, the investigation of causal relationships between these variables was not possible. In this regard, a large-scale longitudinal study is required in the future to investigate the effects of interventions for enhancing CS.

Despite these limitations, the significant contribution of this study is that it determined the level of PSMA for shift nurses working in four general hospitals and confirmed the importance of CS in the relationship between burnout and PSMA among shift nurses.

## 5. Conclusions

This study was conducted to investigate the levels of burnout, CS, and PSMA among nurses on shift work in general hospitals and to examine the mediating effect of CS on the relationship between burnout and PSMA.

The results revealed that CS serves as a partial mediator in burnout and PSMA. The higher the level of burnout among shift nurses, the lower the PSMA. Furthermore, high levels of CS reduce burnout, which leads to an increase in PSMA. Consequently, specific and practical interventions that can minimize job stress are needed, such as the improvement of the nursing work environment and compensation for appropriate treatment for those struggling with special situations, such as the COVID-19 pandemic. A nursing effort at the individual level is needed to increase CS and reduce burnout. In addition, further studies are required on strategies to improve CS among shift nurses as well as the effects of applying the developed strategies.

## Figures and Tables

**Table 1:** Differences in burnout, CS, and PSMA according to general and job-related characteristics of shift-working nurses (*N* = 301).

Characteristics	Categories	*n* (%)	Burnout		CS		PSMA	
			M ± SD	t/F (*p*) Tukey	M ± SD	t/F (*p*) Tukey	M ± SD	t/F (*p*) Tukey
Age (year)	<25	85 (28.2)	2.94 ± 0.56	0.61 (0.545)	2.91 ± 0.67	1.30 (0.274)	4.02 ± 0.56	0.12 (0.885)
	25–<30	120 (39.9)	3.02 ± 0.50		2.93 ± 0.67		3.98 ± 0.59	
	≥30	96 (31.9)	3.00 ± 0.49		3.05 ± 0.66		4.00 ± 0.49	
Gender	Male	18 (6.0)	2.80 ± 0.64	−1.61 (0.109)	3.02 ± 0.72	0.37 (0.713)	4.21 ± 0.63	1.71 (0.089)
	Female	283 (94.0)	3.00 ± 0.51		2.96 ± 0.65		3.98 ± 0.54	
Marital status	Single	238 (79.1)	3.00 ± 0.53	0.80 (0.425)	2.92 ± 0.65	−2.38 (0.018)	3.99 ± 0.57	−0.32 (0.753)
	Married	63 (20.9)	2.94 ± 0.47		3.13 ± 0.65		4.02 ± 0.48	
Religion	No	182 (60.5)	3.04 ± 0.52	2.00 (0.047)	2.87 ± 0.65	−3.08 (0.002)	3.96 ± 0.55	−1.63 (0.104)
	Yes	119 (39.5)	2.92 ± 0.50		3.10 ± 0.65		4.06 ± 0.54	
Education	Diploma ^a^	123 (40.9)	3.05 ± 0.48	3.14 (0.045) *c < a*	2.87 ± 0.63	3.39 (0.035) *a < c*	3.94 ± 0.56	0.89 (0.413)
	Bachelor ^b^	174 (57.8)	2.96 ± 0.53		3.00 ± 0.67		4.03 ± 0.54	
	≥Masters ^c^	4 (1.3)	2.50 ± 0.14		3.60 ± 0.50		4.13 ± 0.30	
Regular exercise	No	129 (42.9)	3.13 ± 0.53	3.41 (0.001)	2.88 ± 0.65	−1.97 (0.050)	3.91 ± 0.51	−2.36 (0.019)
	Yes	172 (57.1)	2.91 ± 0.48		3.03 ± 0.65		4.06 ± 0.57	
Subjective health status *	Good ^a^	80 (26.6)	2.77 ± 0.45	10.96 (<0.001) *a < b,c*	3.12 ± 0.71	3.61 (0.028) n/a	4.12 ± 0.57	2.73 (0.067)
	Moderate ^b^	192 (63.8)	3.06 ± 0.53		2.89 ± 0.60		3.95 ± 0.53	
	Poor ^c^	29 (9.6)	3.14 ± 0.44		3.01 ± 0.76		3.95 ± 0.55	
Clinical Experience (year)	<2	116 (38.5)	2.95 ± 0.56	0.59 (0.555)	2.87 ± 0.62	1.93 (0.171)	4.04 ± 0.56	0.52 (0.593)
	2~<5	79 (26.2)	3.02 ± 0.49		3.02 ± 0.70		3.96 ± 0.58	
	≥5	106 (35.2)	3.01 ± 0.49		3.02 ± 0.66		3.98 ± 0.51	
Position	Staff nurse	288 (95.7)	2.99 ± 0.51	0.69 (0.490)	2.95 ± 0.64	−2.05 (0.042)	4.00 ± 0.54	0.21 (0.832)
	Charge nurse	13 (4.3)	2.89 ± 0.71		3.32 ± 0.83		3.97 ± 0.65	
Working unit *	Medical ward ^a^	91 (30.2)	3.15 ± 0.52	3.13 (0.009) *d < a*	2.86 ± 0.69	0.91 (0.472)	3.96 ± 0.61	1.47 (0.210)
	Surgical ward ^b^	80 (26.6)	2.98 ± 0.57		2.95 ± 0.74		3.98 ± 0.57	
	ICU ^c^	33 (11.0)	2.94 ± 0.44		3.00 ± 0.56		4.24 ± 0.46	
	ED ^d^	33 (11.0)	2.79 ± 0.51		3.10 ± 0.65		3.98 ± 0.47	
	Ped ward/DR	37 (12.3)	2.94 ± 0.44		3.06 ± 0.51		4.00 ± 0.48	
	etc.	27 (9.0)	2.88 ± 0.42		2.99 ± 0.66		3.93 ± 0.50	
Number of night duties (Monthly)	≤ 6	102 (22.9)	2.96 ± 0.60	0.44 (0.727)	2.97 ± 0.73	0.66 (0.580)	3.96 ± 0.59	0.48 (0.700)
	7~8	140 (49.8)	3.00 ± 0.52		2.923 ± 0.67		4.04 ± 0.53	
	≥ 9	49 (16.3)	3.01 ± 0.50		3.07 ± 0.72		3.96 ± 0.52	
Subjective job stress *	Heavy ^a^	198 (65.8)	3.13 ± 0.51	28.67 (<0.001) *c < b< a*	2.88 ± 0.68	5.13 (0.006) n/a	3.94 ± 0.55	6.01 (0.003) *a,b < c*
	Moderate ^b^	91 (30.2)	2.77 ± 0.37		3.10 ± 0.60		4.06 ± 0.55	
	Light ^c^	12 (4.0)	2.36 ± 0.54		3.27 ± 0.36		4,45 ± 0.32	

M, mean; SD, standard deviation; CS, compassion satisfaction; PSMA, patient safety management activities; ICU, intensive care unit; ED, emergency department; DR, delivery room. * Tukey’s Honest Significant Difference ***test*** was used for the post-hoc test.

**Table 2:** Levels of burnout, CS, and PSMA of shift nurses (*N* = 301).

Variables (Item Range)	M ± SD	Range
		Min−Max
Burnout (10−50)	29.89 ± 5.16	12–49
CS (10−50)	29.61 ± 6.55	11–50
PSMA (46−230)	183.92 ± 25.22	108–230

M, mean; SD, standard deviation; CS, compassion satisfaction; PSMA, patient safety management activities.

**Table 3:** Correlations among burnout, CS, and PSMA (*N* = 301).

Variables	Burnout r (*p*)	CS r (*p*)	PSMA r (*p*)
Burnout	1		
CS	−0.66 (<0.001)	1	
PSMA	−0.32 (<0.001)	0.32 (<0.001)	1

CS, compassion satisfaction; PSMA, patient safety management activities.

**Table 4:** Mediating effect of CS on the relationship between burnout and PSMA (*N* = 301).

Variables	B (SE)	β	t (*p*)	R2 (Adj.R2)	F (*p*)
Step 1. Burnout → CS	−0.84 (0.055)	−0.66	−15.16 (<0.001)	0.43 (0.43)	229.66 (<0.001)
Step 2. Burnout → PSMA	−0.34 (0.058)	−0.32	−5.74 (<0.001)	0.10 (0.10)	32.94 (<0.001)
Step 3. Burnout, CS → PSMA					
Burnout → PSMA	−0.20 (0.077)	−0.18	−2.55 (0.011)	0.11 (0.12)	20.64 (<0.001)
CS → PSMA+	0.17 (0.060)	0.20	2.76 (0.006)		
Sobel test: Z = −3.21, *p* = 0.001					

CS, compassion satisfaction; PSMA, patient safety management activities.SE, standard error; Adj, adjusted.

## Data Availability

Data are contained within the article.
